# Crystals of SctV from different species reveal variable symmetry for the cytosolic domain of the type III secretion system export gate

**DOI:** 10.1107/S2053230X22009736

**Published:** 2022-10-14

**Authors:** Dominic Gilzer, Eileen Baum, Nele Lieske, Julia L. Kowal, Hartmut H. Niemann

**Affiliations:** a Department of Chemistry, Bielefeld University, Universitätsstrasse 25, 33615 Bielefeld, Germany; European Molecular Biology Laboratory, France

**Keywords:** type III secretion systems, export-gate protein SctV, *Photorhabdus luminescens*, *Aeromonas hydrophila*, cyclic oligomers, low-resolution crystallography, molecular replacement, oligomerization, self-rotation function

## Abstract

Type III secretion systems (T3SSs) are proteinaceous devices employed by Gram-negative bacteria to directly transport proteins into a host cell. Substrate recognition and secretion are strictly regulated by the export apparatus of the so-called injectisome. The export gate SctV engages chaperone-bound substrates of the T3SS in its nonameric cytoplasmic domain. Here, the purification and crystallization of the cytoplasmic domains of SctV from *Photorhabdus luminescens* (LscV_C_) and *Aeromonas hydrophila* (AscV_C_) are reported. Self-rotation functions revealed that LscV_C_ forms oligomers with either eightfold or ninefold symmetry in two different crystal forms. Similarly, AscV_C_ was found to exhibit tenfold rotational symmetry. These are the first instances of SctV proteins forming non-nonameric oligomers.

## Abstract 2

Three crystal forms of the type III secretion system export-gate protein SctV from two bacterial species reveal different cyclic oligomers, namely an octamer, a nonamer and a decamer.

## 1. Introduction

Several Gram-negative bacteria, including the human pathogens *Yersinia*, *Salmonella* and *Shigella*, employ a type III secretion system (T3SS) as a tool to evade the immune response of the host or to induce cytotoxicity (Coburn *et al.*, 2007 ▸). The T3SS is anchored in both bacterial membranes via its basal body and contacts the host cell with its protruding needle structure. Hydrophobic translocator proteins insert themselves into the host cell membrane, thereby forming a continuous channel from the bacterial cytoplasm to the host cytoplasm (Portaliou *et al.*, 2016 ▸). Since effector proteins are transported directly into the host cell, virulent T3SSs are also termed injectisomes. Many of the proteins involved in injecti­somes have homologs within the T3SS of the bacterial flagellum, as the two systems are likely to share an evolutionary ancestor.

One of these conserved structures within the T3SS is the export apparatus, a complex containing five different protein species, one of which is the export-gate protein SctV or FlhA in flagella. SctVs are ∼77 kDa proteins comprising an N-terminal transmembrane anchor followed by an ∼40 kDa cytosolic domain (SctV_C_). Structurally, the transmembrane domain remains largely uncharacterized. It has been shown that secretion is powered by the proton motive force (Minamino & Namba, 2008 ▸) and that protonation of the cytosolic PHIPEP region within the transmembrane domain of FlhA triggers larger conformational changes that also affect FlhA_C_ (Erhardt *et al.*, 2017 ▸). The cytoplasmic domain recognizes T3S substrates, which are usually escorted by a cognate chaperone. Ternary-complex structures of the export gate with a substrate–chaperone pair have revealed different binding modes in flagellar (Xing *et al.*, 2018 ▸) and injectisomal (Gilzer *et al.*, 2022 ▸) T3SSs. Recognition by the export gate is mediated by either the chaperone or the substrate, respectively.

Oligomerization of SctV_C_/FlhA_C_ has been observed *in vivo* using fluorescence microscopy (Diepold *et al.*, 2017 ▸; Li & Sourjik, 2011 ▸; Morimoto *et al.*, 2014 ▸) and *in situ* electron tomography (Butan *et al.*, 2019 ▸; Hu *et al.*, 2017 ▸), but the exact stoichiometry of the export gate could not be determined. Based on published structures of SctV_C_ and FlhA_C_, the proteins are expected to form cyclic nonamers in the secretion system (Abrusci *et al.*, 2013 ▸; Majewski *et al.*, 2020 ▸; Jensen *et al.*, 2020 ▸; Matthews-Palmer *et al.*, 2021 ▸; Xu *et al.*, 2021 ▸; Kuhlen *et al.*, 2021 ▸; Yuan *et al.*, 2021 ▸; Gilzer *et al.*, 2022 ▸). Monomeric structures have been described and show a similar fold to the nonameric state (Saijo-Hamano *et al.*, 2010 ▸; Moore & Jia, 2010 ▸; Bange *et al.*, 2010 ▸; Worrall *et al.*, 2010 ▸; Xing *et al.*, 2018 ▸) with four subdomains (SD1–SD4) arranged in an U shape. In general, nonamerization is mediated via the highly conserved SD3 of SctV_C_ as well as SD1. The linker connecting the cytoplasmic domain to the transmembrane domain binds a groove of the adjacent protomer in the ring and thereby stabilizes the nonamer (Kuhlen *et al.*, 2021 ▸).

Here, we report the purification and crystallization of the *Photorhabdus luminescens* and *Aeromonas hydrophila* SctV proteins (LscV and AscV, respectively). We obtained crystals of the cytoplasmic domain of LscV (LscV_C_) alone as well as of LscV_C_ and AscV_C_ in complex with a substrate–chaperone pair. The self-rotation functions revealed that LscV_C_ is able to adopt either a nonameric or octameric rotational symmetry and that AscV_C_ can incorporate an additional tenth protomer into the cyclic assembly.

## 2. Materials and methods

### 2.1. Protein expression and purification

The cytosolic domain of LscV (LscV_C_; residues 357–705) was cloned from genomic *P. luminescens* DNA into pETM-11 vector (for further details, see Table 1 ▸). For protein production, *Escherichia coli* BL21 (DE3) cells were grown at 310 K in LB medium containing 30 µg ml^−1^ kanamycin to an OD_600_ of approximately 0.5. The temperature was then reduced to 293 K and expression of His_6_-LscV_C_ was induced at an OD_600_ of ∼0.8 using 0.25 m*M* isopropyl β-d-1-thio­galactopyranoside (IPTG). After incubation at 293 K overnight, the cells were pelleted at 4600*g* and resuspended in ice-cold lysis buffer (50 m*M* Tris–HCl pH 8.0, 150 m*M* NaCl, 10 m*M* β-mercaptoethanol) supplemented with 0.6 mg DNase I per litre of culture as well as a cOmplete protease-inhibitor cocktail tablet (Roche). Lysis using a Stansted FPG12800 pressure-cell homogenizer (120 MPa) was followed by centrifugation (60 min, 30 000*g*, 297 K).

The supernatant was supplemented with 10 m*M* imidazole and applied onto 8 ml Protino Ni–NTA agarose resin (Macherey-Nagel). Incubation took place at 297 K for 1 h before the flowthrough was collected. Washing the column with wash buffer [20 m*M* Tris–HCl pH 8.0, 300 m*M* NaCl, 1 m*M* dithiothreitol (DTT) and 30 m*M* followed by 100 m*M* imidazole] ensured the elution of weakly bound impurities. The target protein was eluted using elution buffer (20 m*M* Tris–HCl pH 8.0, 150 m*M* NaCl, 1 m*M* DTT, 250 m*M* imidazole) and dialyzed against 2 × 2 l dialysis buffer (20 m*M* Tris–HCl pH 8.0, 150 m*M* NaCl, 1 m*M* DTT) overnight after adding 1:50(*w*:*w*) TEV protease to the protein solution to remove the affinity tag. Residual His_6_-LscV_C_ was removed by a second Ni–NTA affinity-chromatography step using 5 ml of resin. Afterwards, the flowthrough and wash fractions from the second affinity-chromatography step were applied onto 7 ml Source 15Q anion-exchange resin packed into a Tricorn 10/100 column (Cytiva) and eluted using a gradient from 20 m*M* Tris–HCl pH 8.0 to 20 m*M* Tris–HCl pH 8.0, 1 *M* NaCl. As a final step, the buffer was exchanged to 20 m*M* Tris–HCl pH 8.0, 150 m*M* NaCl by size-exclusion chromatography (SEC) using a HiLoad 16/60 Superdex 200 prep-grade (Cytiva) column. LscV_C_ was frozen with 5 m*M* tris(2-carboxyethyl)phosphine (TCEP).

Similarly, the cytosolic domain of *A. hydrophila* AscV (AscV_C_; residues 375–721) was cloned into pETM-11 for expression as an N-terminally hexahistidine-tagged protein (Table 1 ▸). Expression and lysis were carried out as described for LscV_C_, but a HisTrap HP (1 ml; Cytiva) column was used for protein capture. The cleared lysate was applied onto the column and unbound protein was washed off using binding buffer (50 m*M* Tris–HCl pH 8.0, 500 m*M* NaCl, 1 m*M* DTT, 30 m*M* imidazole). Elution was performed via a gradient to elution buffer (50 m*M* Tris–HCl pH 8.0, 500 m*M* NaCl, 1 m*M* DTT, 300 m*M* imidazole) over 30 ml. Subsequently, TEV digestion and a second Ni–NTA affinity-chromatography step were carried out as before. Ion-exchange chromatography was unnecessary due to the higher purity of AscV_C_. Instead, SEC was used after the second affinity-chromatography step following the same protocol as for LscV_C_.

The YscX_32_–YscY and AscX_31_–YscY substrate–chaperone complexes were expressed as MBP-YscX_32_/MBP-AscX_31_ and His_6_-YscY (Table 1 ▸) and were prepared largely as described previously for YscX–YscY (Gilzer *et al.*, 2022 ▸), but changing the gravity-flow amylose affinity chromatography to a high-flow setup. Here, 8 ml Amylose Resin High Flow (New England Biolabs) was packed into a Tricorn 10/100 column (Cytiva). The cleared lysate was applied onto the column and unbound protein was washed off using amylose wash buffer (50 m*M* Tris–HCl pH 8.0, 200 m*M* NaCl, 1 m*M* EDTA, 10 m*M* β-mercaptoethanol). Addition of 10 m*M* maltose to the buffer resulted in elution of the MBP-tagged target protein. TEV digestion was carried out to remove the MBP tag. Afterwards, Ni–NTA affinity-chromatography and SEC via a HiLoad 16/60 Superdex 75 prep-grade (Cytiva) column were used to further purify the complex.

### 2.2. Crystallization

Initial screens were set up at 277 and 295 K using a Crystal Gryphon pipetting robot (Art Robbins Instruments) and commercially available crystallization screens. For LscV_C_ at 5 mg ml^−1^, various conditions containing sulfate or phosphate salts yielded intergrown crystals within three days. Crystal growth was improved in the optimized conditions summarized in Table 2 ▸. For cryoprotection, LscV_C_ crystals were transferred to a solution supplemented with 20%(*v*/*v*) glycerol.

Reconstitution of the ternary complex containing LscV_C_, YscX_32_ and YscY was achieved by mixing the proteins in an equimolar fashion 2 h prior to setting up the crystallization plates. Initial hits were obtained in 0.1 *M* HEPES pH 7.0, 1.0 *M* succinic acid, 1%(*w*/*v*) PEG 2000 MME and were not optimized further (Table 2 ▸). Due to the fragility of the crystals, cryoprotection was carried out by transferring the crystals first to reservoir solution containing 10%(*v*/*v*) propylene glycol and then to reservoir solution containing 20%(*v*/*v*) propylene glycol.

The ternary complex of AscV_C_, AscX_31_ and YscY was reconstituted by incubating an equimolar mixture of the proteins for 2 h on ice before plate setup. The initial hits for this complex were spherulites that were obtained in 1.6 *M* sodium/potassium phosphate pH 7.0, which could be optimized to 1.4 *M* sodium/potassium phosphate pH 7.0 (see Table 2 ▸). AscV_C_–AscX_31_–YscY crystals were cryoprotected in reservoir solution with 22.5%(*v*/*v*) glycerol.

### 2.3. Data collection and processing

Diffraction data were collected using the local installations of *MXCuBE*2 (Oscarsson *et al.*, 2019 ▸) or *MXCuBE*3 on beamlines P14 (LscV_C_) at DESY, Hamburg, Germany and ID23-1 (LscV_C_–YscX_32_–YscY) and ID30B (AscV_C_–AscX_31_–YscY) at ESRF, Grenoble, France (Mueller-Dieckmann *et al.*, 2015 ▸). *XDS* (Kabsch, 2010 ▸) was used for processing via *XDSGUI* and scaling was carried out using *XSCALE*. Merged data were used in all subsequent steps. Anisotropy was determined with the *STARANISO* server (Tickle *et al.*, 2018 ▸). The solvent content was estimated with *phenix.xtriage* (Zwart *et al.*, 2005 ▸; Liebschner *et al.*, 2019 ▸). Self-rotation functions were generated with *MOLREP* (Vagin & Teplyakov, 2010 ▸) within the *CCP*4 suite (Winn *et al.*, 2011 ▸) without applying a resolution cutoff. Molecular replacement was performed in *Phaser* (McCoy *et al.*, 2007 ▸) and rigid-body refinement was performed in *phenix.refine* (Afonine *et al.*, 2012 ▸).

## 3. Results

**Figure 1:**
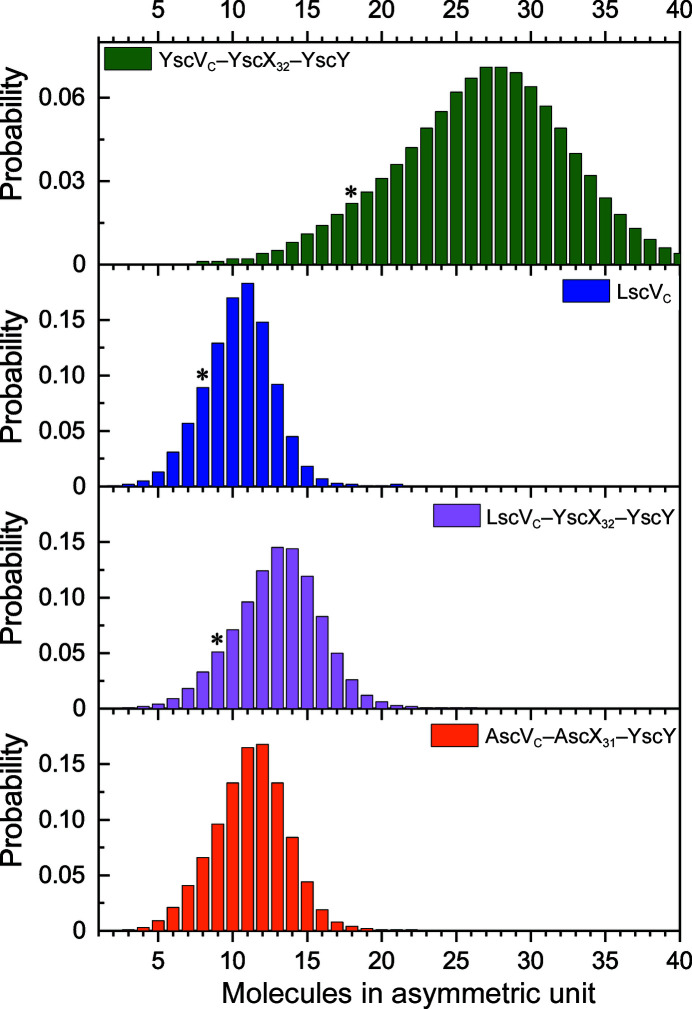
Solvent-content analysis. Probabilities for different compositions of the asymmetric unit were calculated using *phenix.xtriage*. Copy numbers that were confirmed via MR are marked with an asterisk (*).

T3SS export gates have been shown to form cyclic nonamers via their cytoplasmic domains (Abrusci *et al.*, 2013 ▸; Majewski *et al.*, 2020 ▸; Jensen *et al.*, 2020 ▸; Matthews-Palmer *et al.*, 2021 ▸; Xu *et al.*, 2021 ▸; Kuhlen *et al.*, 2021 ▸; Yuan *et al.*, 2021 ▸; Gilzer *et al.*, 2022 ▸). We purified the cytosolic domain of *P. luminescens* LscV (LscV_C_) and successfully crystallized it using 0.1 *M* Tris–HCl pH 8.0, 1.3 *M* ammonium sulfate at 293 K. Unfortunately, the purification of LscV_C_ could not be reproduced. Low-resolution data were obtained to approximately 4.1 Å according to *I*/σ(*I*) ≃ 2, and processing in *XDS* (Kabsch, 2010 ▸) revealed that the protein crystallized in space group *P*2_1_2_1_2 with a large unit cell that could accommodate an oligomeric assembly in its asymmetric unit (Table 3 ▸). Correspondingly, solvent-content analysis in *phenix.xtriage* (Zwart *et al.*, 2005 ▸; Liebschner *et al.*, 2019 ▸) confirmed the presence of multiple copies in the asymmetric unit, with 11 molecules per asymmetric unit as the most likely option (Fig. 1 ▸). A similar overestimation of the copy number in the asymmetric unit was observed for our previously published structure of the *Yersinia* export gate bound to the YscX–YscY substrate–chaperone complex (Gilzer *et al.*, 2022 ▸), where 28 copies of each molecule were estimated but only two nonamers were present in the asymmetric unit (Fig. 1 ▸). Despite the high sequence conservation, with 81% identity between LscV_C_ and YscV_C_, molecular-replacement (MR) trials employing the nonameric ring of YscV_C_ (PDB entry 7alw; Kuhlen *et al.*, 2021 ▸) as the search model failed.

**Figure 2:**
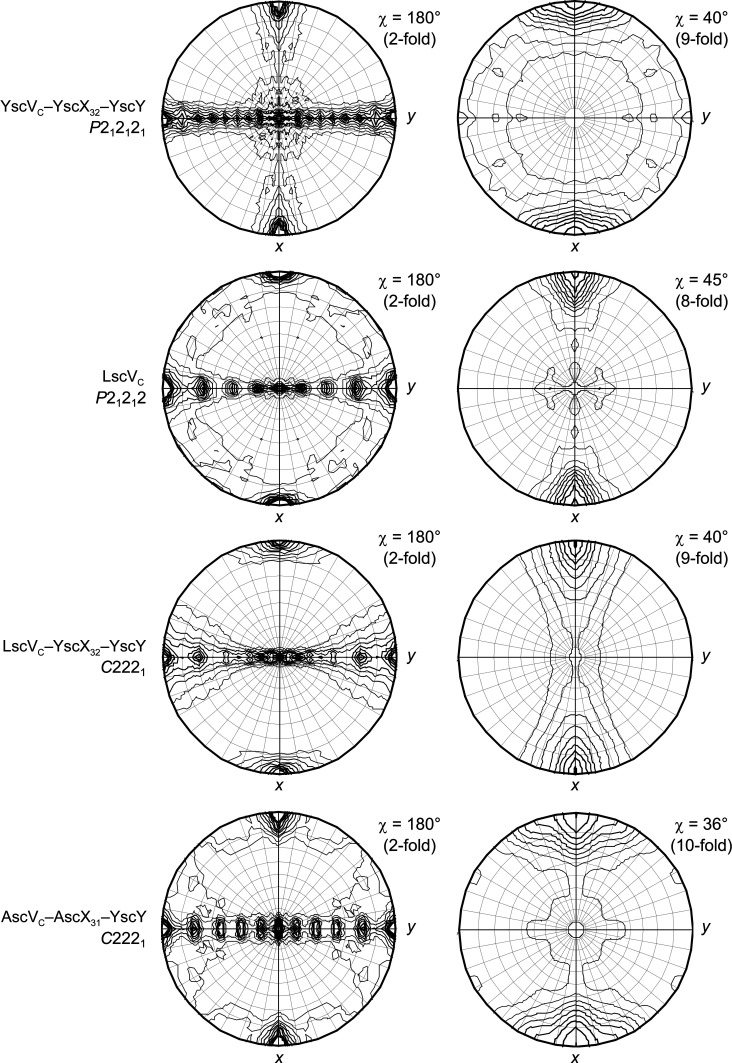
Self-rotation functions of YscV_C_–YscX_32_–YscY, LscV_C_, LscV_C_–YscX_32_–YscY and AscV_C_–AscX_31_–YscY (from top to bottom) calculated by *MOLREP* without applying a high-resolution cutoff. Sections at χ = 180° reveal a planar arrangement of multiple twofold axes. A perpendicular ninefold, eightfold or tenfold symmetry can be observed in the corresponding χ sections for the three proteins.

To further investigate this discrepancy between a high degree of homology to YscV_C_ and our unsuccessful attempts to employ it as search model for LscV_C_, we calculated self-rotation functions (SRF) in *MOLREP*. For the *Yersinia* ternary complex YscV_C_–YscX_32_–YscY, the SRF at χ = 180° shows 18 peaks in one plane and an additional peak perpendicular to it (Fig. 2 ▸). This behavior is caused by the stacking of two nonameric rings within the asymmetric unit of YscV_C_–YscX_32_–YscY, which results in 18 noncrystallographic twofold rotational axes along the nonamer–nonamer interface. Dimers of SctV_C_ nonamers have been reported previously and were observed to stack either via the membrane-proximal (Majewski *et al.*, 2020 ▸; Xu *et al.*, 2021 ▸; Yuan *et al.*, 2021 ▸) or membrane-distal (Kuhlen *et al.*, 2021 ▸; Gilzer *et al.*, 2022 ▸) side. Interestingly, the SRF of LscV_C_ shows only eight peaks in the same plane for χ = 180°, suggesting the presence of only eight molecules in the asymmetric unit (Fig. 2 ▸). In fact, MR was successful and produced a single solution when searching for eight consecutive YscV_C_ monomers in *Phaser* (McCoy *et al.*, 2007 ▸), generating a single solution with a TFZ = 26.6 and eLLG = 1602. The placement of a ninth copy of the search model was not successful as it resulted in severe clashing with previously placed copies. This is reflected in the TFZ values, which increase with the number of monomers placed to TFZ = 25.9 for the eighth copy, but decrease sharply to TFZ = 5.7 for the ninth molecule (Supplementary Table S1). Within the LscV_C_ crystal, symmetry-related cyclic octamers stack onto each other via their membrane-distal sides, resulting in the peaks seen in the SRF. The eightfold axis runs parallel to the *a* axis of the unit cell and is perpendicular to the *bc* plane (Supplementary Fig. S1). Some clashes occur at the interface of two stacked oligomers and at the closest point between laterally adjacent octamers (Supplementary Fig. S2). The electron density is considerably weaker when compared with the surrounding regions, suggesting local rearrangements or rigid-body movements of subdomains when compared with the search model. Subdomain SD2, which is involved in one clash and has poor density in the LscV_C_ structure, is also particularly flexible in other SctV proteins and has been suggested to undergo rigid-body movements (Yuan *et al.*, 2021 ▸). Initial rigid-body refinement in *phenix.refine* resulted in *R*_work_ = 0.4593 and *R*_free_ = 0.4482, indicating that the overall placement is correct.

**Figure 3:**
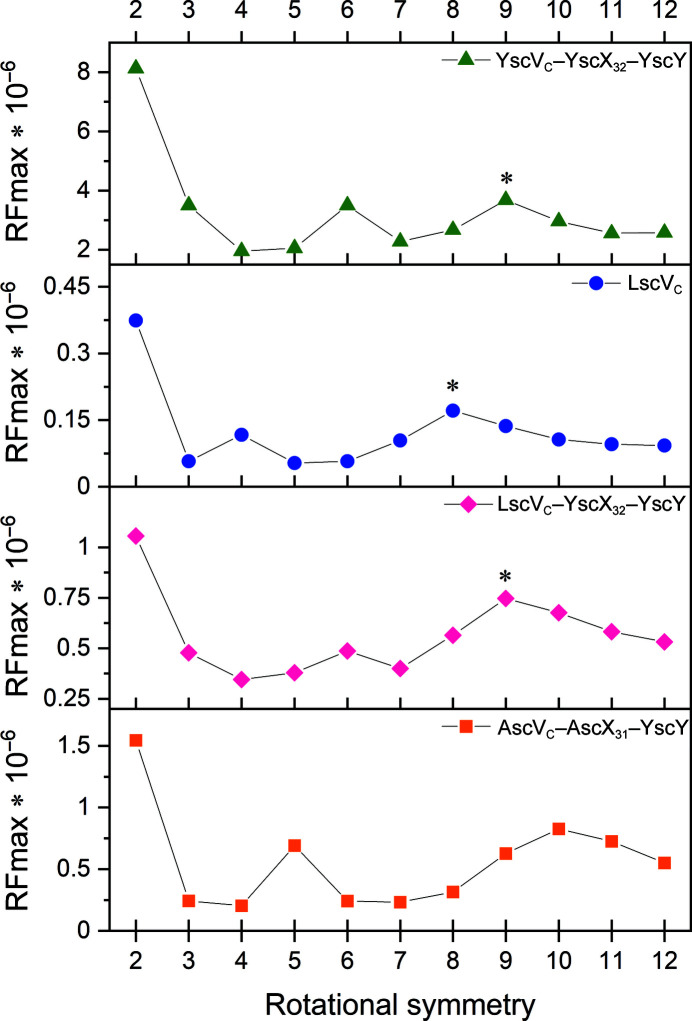
Peak height of self-rotation functions for YscV_C_–YscX_32_–YscY, LscV_C_, LscV_C_–YscX_32_–YscY and AscV_C_–AscX_31_–YscY. All calculations were performed in *MOLREP* for χ sections corresponding to twofold to 12-fold rotational symmetries. An asterisk (*) marks the rotational symmetry of the cyclic oligomer observed after molecular replacement.

We later obtained a different crystal form containing LscV_C_ co-crystallized with an independently purified substrate–chaperone complex. The new crystal form gave us the opportunity to check whether the octameric stoichiometry is a genuine difference in the oligomerization states between species. During our attempts to purify binary substrate–chaperone complexes with the substrate SctX from *P. luminescens* (LscX), LscX_31_–LscY and LscX_31_–YscY formed a heavy precipitate upon concentrating the proteins. Therefore, we instead generated a heterologous complex of LscV_C_ and the *Y. enterocolitica* substrate YscX_32_ and chaperone YscY. The ability of these T3SS proteins to produce heterologous binary as well as ternary complexes with export gates had previously been established (Gurung *et al.*, 2018 ▸). The proteins were mixed and incubated for 2 h before setting up crystallization plates to allow formation of the ternary complex. Crystals were obtained but only diffracted to approximately 8 Å resolution. Data processing in *XDS* revealed a large unit cell similar to that of the published YscV_C_–YscX_32_–YscY complex (Table 3 ▸; Gilzer *et al.*, 2022 ▸), which crystallized in space group *P*2_1_2_1_2_1_ with unit-cell parameters *a* = 143.46, *b* = 324.92, *c* = 369.38 Å. The new crystal form of LscV_C_–YscX_32_–YscY belongs to the related space group *C*222_1_, with unit-cell parameters *a* = 138.49, *b* = 372.64, *c* = 324.65 Å. In fact, the condition in which this crystal was obtained is identical to the initial hit from which the *Yersinia* ternary-complex crystals were obtained. Analysis of the solvent content in *phenix.xtriage* suggested a composition of 13 molecules per asymmetric unit (Fig. 1 ▸). In contrast to LscV_C_ alone, the SRF at χ = 180° suggested a cyclic nonamer (Fig. 2 ▸), as was underlined by higher RFmax values for threefold, sixfold and ninefold rotational symmetry axes compared with fourfold and eightfold axes (Fig. 3 ▸). An attempt to solve the structure by searching for nine heterotrimeric YscV_C_–YsX_32_–YscY complexes extracted from PDB entry 7qij (Gilzer *et al.*, 2022 ▸) produced no solution. However, molecular replacement was successful when employing either a YscV_C_ nonamer (PDB entry 7alw; TFZ = 20.4; eLLG = 348) or the nonameric YscV_C_–YscX_32_–YscY complex (PDB entry 7qij; TFZ = 31.2; eLLG = 884) as a search model. Searching for PDB entry 7alw, an EM structure that obeys strict *C*_9_ symmetry, generated a single solution. Using PDB entry 7qij, a crystal structure with noncrystallographic ninefold pseudo-symmetry, as a model resulted in nine solutions that were related to each other by rotation around the ninefold axis. From rigid-body refinement in *phenix.refine*, *R*_work_ = 0.3642 and *R*_free_ = 0.3552 for PDB entry 7qij and *R*_work_ = 0.4624 and *R*_free_ = 0.4703 for PDB entry 7alw were obtained. The global placement of the complex is therefore correct with LscV_C_ arranged as a cyclic nonamer. When compared with the homologous YscV_C_ complex the packing is identical, with most crystal contacts formed between YscY molecules. Only one LscV_C_–YscX_32_–YscY nonamer is present in the asymmetric unit, compared with two rings in the YscV_C_–YscX_32_–YscY asymmetric unit since the *C*-centering caused the conversion of a twofold NCS into a crystallographic symmetry operator (Supplementary Fig. S3).

Furthermore, we purified and crystallized the cytosolic domain of the *A. hydrophila* T3SS export gate (AscV_C_). While crystals grew readily, the resulting data were of poor quality due to a combination of low resolution and smeared reflections. Consequently, we attempted to co-crystallize AscV_C_ with the substrate–chaperone complex AscX_31_–YscY by co-incubating the protein for 2 h before crystallization screens were set up. Crystals of this complex diffracted poorly to around 7–8 Å resolution, but the data could be processed using *XDS* in space group *C*222_1_ with a unit cell that was large enough to fit a cyclic oligomer (Table 3 ▸). Interestingly, the AscV_C_–AscX_31_–YscY complex showed almost no anisotropy, while the diffraction of three other SctV-containing crystals [YscV_c_–YscX_32_–YscY (PDB entry 7qij), LscVc and LscV_C_–YscX_32_–YscY] was severely anisotropic (Supplementary Table S2). Initial analysis in *phenix.xtriage* suggested that the asymmetric unit probably contains 12 molecules (Fig. 1 ▸). The SRF, however, revealed ten coplanar maxima for χ = 180° (Fig. 2 ▸), indicating that ten molecules are present in the asymmetric unit. An MR search for nine copies of either YscV_C_ or the heterotrimeric YscV_C_–YscX_32_–YscY complex was not successful. This is not surprising given the fact that the same approach had also failed for the LscV_C_–YscX_32_–YscY complex, which diffracted to the same resolution but has slightly worse data quality. We also searched for nine or ten copies of modified search models, namely YscV_C_ from PDB entry 7alw truncated by *phenix.sculptor* according to the Schwarzenbacher algorithm or truncated to a C^α^ model and an *AlphaFold*2 (Jumper *et al.*, 2021 ▸) model of AscV_C_. All of these attempts produced incorrect solutions with TFZ values between 6.4 and 7.7, clashes between monomers and monomers not arranged as rings. The placement of a nonameric ring using YscV_C_ (PDB entry 7alw) resulted in TFZ = 7.7 and eLLG = 26, which again indicates an incorrect solution to the phase problem. This was underlined by poor electron density produced in this MR and severe clashing, resulting in a near-complete overlap of nonameric rings and large gaps between assemblies along the ninefold symmetry axis (Supplementary Fig. S4). Searching for a nonameric ring of YscV_C_–YscX_32_–YscY (PDB entry 7qij) was also not successful, as no solution passed the packing function.

To establish whether the ten peaks in the self-rotation function of AscV_C_–AscX_31_–YscY can be attributed to a cyclic decamer, as was the case for the LscV_C_ octamer, we calculated SRFs for all possible rotational symmetries between twofold and 12-fold in *MOLREP* without applying a high-resolution cutoff (Fig. 3 ▸). The maxima of the SRFs calculated for the AscV_C_-containing complex in the χ = 72° (fivefold rotational symmetry) and at χ = 36° (tenfold) sections are higher than for the surrounding χ values. Conversely, fourfold and eightfold axes were favored when data from the octameric LscV_C_ were analyzed. Truncating the LscV_C_ data to 7.0 Å resolution (the same resolution as AscV_C_–YscX_32_–YscY) does not change the appearance of the SRFs, but only changes the RFmax values slightly. Nevertheless, in SRFs of LscV_C_ calculated with a high-resolution limit of 7.0 Å, the RFmax for an eightfold rotation remains higher than the RFmax for sevenfold or ninefold axes (data not shown). For the nonameric YscV_C_–YscX_32_–YscY complex, threefold, sixfold and ninefold symmetries appear as peaks (Fig. 3 ▸). Given the behavior observed for the LscV_C_ octamer, a cyclic AscV_C_ decamer that stacks onto a symmetry-related decamer would explain the SRF of AscV_C_–AscX_31_–YscY. The corresponding composition of ten molecules in the asymmetric unit agrees with the results from *phenix.xtriage*.

## 4. Discussion

Variable symmetries are not unprecedented for protein complexes with high orders of rotational symmetry and have been observed, for instance, for secretins (Bayan *et al.*, 2006 ▸). Cryo-EM of the rotor of the flagellar motor showed variable rotational symmetries for the M ring (24-fold to 26-fold) and the C ring (32-fold to 36-fold) (Thomas *et al.*, 2006 ▸). The inner membrane ring of the *Salmonella typhimurium* type III secretion needle complex revealed 19-fold to 22-fold symmetry in initial EM analysis (Marlovits *et al.*, 2004 ▸, 2006 ▸). Later cryo-EM structures showed (pseudo-)24-fold rotational symmetry for the inner membrane ring of needle complexes from *S. typhimurium* and *Shigella flexneri* (Hodgkinson *et al.*, 2009 ▸; Schraidt & Marlovits, 2011 ▸).

In crystallography, the use of SRFs to establish the order of rotation for cyclic or dihedral oligomers is widely accepted (Schoch *et al.*, 2015 ▸; Matsuno *et al.*, 2015 ▸). Bacteriophage portal proteins represent an example that is particularly relevant to our work. Portal proteins always insert into the viral head as cylindrical dodecamers. However, overexpressed portal proteins on their own can also assemble into other cyclic oligomers (Cuervo & Carrascosa, 2012 ▸; van Heel *et al.*, 1996 ▸). Cryo-EM of the overexpressed T4 portal protein revealed rings mainly with 12-fold, but also with 11-fold and 13-fold, symmetry. Crystals of this protein sample diffracted to only 6.5 Å resolution. Rossmann and coworkers suggested that the different oligomeric states of the sample might be the main factor that limits the resolution of the crystals (Sun *et al.*, 2015 ▸). Different crystal forms of the T7 portal allowed structure determination with either *C*_12_ or *C*_13_ symmetry (Cuervo *et al.*, 2019 ▸). Using an approach that was basically identical to ours, Coll and coworkers determined the rotational order of the T7 portal in the crystals using the peak height of the SRF at various χ angles and the number of peaks at χ = 180° (Fàbrega-Ferrer *et al.*, 2021 ▸).

While only monomeric or nonameric structures of T3SS export gates have been reported to this point, our results illustrate that both LscV_C_ from *P. luminescens* and AscV_C_ from *A. hydrophila* form non-nonameric cyclic assemblies. Using self-rotation functions, we deduced that LscV_C_ can adopt either an octameric or a nonameric stoichiometry within the crystal environment and consequently solved the phase problem for both crystal forms. By comparing the behavior of AscV_C_ with its homologs, we showed that it presumably decamerizes instead. The oligomeric state of AscV_C_ and LscV_C_ in solution remains unknown. Gel filtration and multi-angle light scattering would most likely not distinguish reliably between octamers, nonamers and decamers because the mass difference is only about 10%. Precise determination of the molecular mass by SEC or light scattering is further complicated by the fact that the oligomerization of SctV_C_ proteins is concentration-dependent. YscV_C_, for example, is mostly monomeric at low concentration (Gilzer *et al.*, 2022 ▸). Moreover, mixtures of different oligomers might exist in solution, as observed for the T4 portal protein (Sun *et al.*, 2015 ▸). One could imagine an equilibrium of LscV_C_ octamers and nonamers in solution. Crystallization of octamers would remove them from solution and cause nonamers to shift to octamers. Other explanations for the different SctV_C_ oligomers are conceivable. It is possible that LscV_C_ on its own forms octamers in solution, while the binding of the YscX_32_–YscY complex induces the formation of nonameric rings. Finally, we cannot exclude that crystal-packing forces cause the deviation from the common *C*_9_ symmetry. However, to the best of our knowledge, the accidental formation of higher order cyclic oligomers in the asymmetric unit of a crystal is a rare event. Hence, it remains to be established whether these non-nonameric assemblies can also form at the export apparatus.

## Supplementary Material

Supplementary Tables and Figures. DOI: 10.1107/S2053230X22009736/ow5033sup1.pdf

## Figures and Tables

**Table 1:** Macromolecule-production information Artificially introduced residues are underlined and TEV protease recognition sites are shown in bold, with a slash indicating the cleaved position. AscV_C_ and AscX_31_–YscY were expressed and purified independently before reconstituting the complex for crystallization.

	LscV_C_	AscV_C_	AscX_31_–YscY	YscX_32_–YscY
Source organism	*Photorhabdus laumondii* TT01	*Aeromonas hydrophila* AH3	*Aeromonas hydrophila* AH3; *Yersinia enterocolitica* W22703	*Yersinia enterocolitica* W22703
DNA source	Genomic DNA	Synthetic	Synthetic; pYVe227 plasmid	pYVe227 plasmid
Expression vector	pETM-11	pETM-11	pETM-40; pACYCDuet-1	pETM-40; pACYCDuet-1
Expression host	*E. coli* BL21 (DE3)	*E. coli* BL21 (DE3)	*E. coli* BL21 (DE3)	*E. coli* BL21 (DE3)
Construct description	His_6_-TEV-LscV_357–705_	His_6_-TEV-AscV_375–721_	MBP-TEV-AscX_31–121_; His_6_-YscY_1–109_	MBP-TEV-YscX_32–121_; His_6_-YscY_1–109_
Complete amino-acid sequence of the construct produced	MKHHHHHHPMSDYDIPTT**ENLYFQ/G** AMAKAGKLSEKEEFAMTVPLLIDVDAGLQAELEAISLNDELIRVRRALYLDLGVPFPGIHLRFNEGMKEGEYLIQLQEVPVARGRLRSAHLLVQEPVSQLELLAIPYEEGEPLLPNQPTLWVAEAHQERLVKSGLAALSMSQVITWHLSHVLREYAEDFIGVQETRYLLEQMEGSYGELVKEAMRIIPLQRMTEILQRLVGEDISIRNTRTILEAMVVWGQKEKDVVQLTEYIRSSLKRYICYKYANGNNILPAYLLDQQVEEQIRGGIRQTSAGSYLALDPAVTQSFLEQMKKTVGDLTQMQNKPVLIVSMDIRRYVRKLIEGDHHGLPVLSYQELTQQINIQPLGRVCL	MKHHHHHHPMSDYDIPTT**ENLYFQ/G** AMARGKLGEKEEFAMTVPLLIDVDAALQADLEAIALNDELVRVRRALYLDLGVPFPGIHLRFNEGMGPGEYLIQLQEVPVARGLLRPGHQLVQENASQLDLLGIPYEEGAPLLPGQPTLWVANEHQDRLEKSRLATLTTGQVVTWHLSHVLREYAEDFIGIQETRYLLEQMEGSYGELVKEAQRIIPLQRMTEILQRLVGEDISIRNMRAILEAMVEWGQKEKDVVQLTEYIRSSLKRYICYKYANGNNILPAYLLDQQVEEQIRGGIRQTSAGSYLALDPTITQGFLDQVRHTVGDLAQMQNKPVLIVSMDIRRYVRKLIEGDYHALPVLSYQELTQQINIQPLGRVCL	MBP-TEV-AscX_31–121_: MBP-NSSSNNNNNNNNNNPMS**ENLYFQ/G** AMALLPDGQSIEPHISRLYPERLADRALLDFATPHRGFHDLLRPVDFHQAMQGLRSVLAEGQSPELRAAAILLEQMHADEQLMQMTLHLLHKV	MBP-TEV-YscX_32–122_: MBP-NSSSNNNNNNNNNNPMS**ENLYFQ/G** AMGALPPDGHPVEPHLERLYPTAQSKRSLWDFASPGYTFHGLHRAQDYRRELDTLQSLLTTSQSSELQAAAALLKCQQDDDRLLQIILNLLHKV
			His_6_-YscY_1–109_: MGHHHHHHGNITLTKRQQEFLLLNGWLQLQCGHAERACILLDALLTLNPEHLAGRRCRLVALLNNNQGERAEKEAQWLISHDPLQAGNWLCLSRAQQLNGDLDKARHAYQHYLELKDHNESP	His_6_-YscY_1–109_: MGHHHHHHGNITLTKRQQEFLLLNGWLQLQCGHAERACILLDALLTLNPEHLAGRRCRLVALLNNNQGERAEKEAQWLISHDPLQAGNWLCLSRAQQLNGDLDKARHAYQHYLELKDHNESP

**Table 2:** Crystallization conditions AscV_C_ and AscX_31_–YscY as well as LscV_C_ and YscX_32_–YscY were mixed in an equimolar fashion and pre-incubated for 2 h prior to plate setup.

	LscV_C_	LscV_C_–YscX_32_–YscY	AscV_C_–AscX_31_–YscY
Method	Sitting-drop vapor diffusion	Sitting-drop vapor diffusion	Sitting-drop vapor diffusion
Plate type	Cryschem M Plate, Hampton Research	MRC 2 Lens Crystallization Plate, SWISSCI	MRC 2 Lens Crystallization Plate, SWISSCI
Temperature (K)	295	295	295
Protein concentration (mg ml^−1^)	5	LscV_C_, 3.1; YscX_32_–YscY, 1.8	AscV_C_, 3.1; AscX_31_–YscY, 1.8
Buffer composition of protein solution	20 m*M* Tris–HCl pH 8.0, 150 m*M* NaCl, 5 m*M* TCEP	20 m*M* Tris–HCl pH 8.0, 150 m*M* NaCl, 2 m*M* TCEP	20 m*M* Tris–HCl pH 8.0, 150 m*M* NaCl, 2 m*M* TCEP
Composition of reservoir solution	0.1 *M* Tris–HCl pH 8.0, 1.3 *M* ammonium sulfate	0.1 *M* HEPES pH 7.0, 1.0 *M* succinic acid, 1%(*w*/*v*) PEG 2000 MME	1.4 *M* sodium/potassium phosphate pH 7.0
Volume of drop (µl)	3	0.3	0.3
Drop ratio (protein:reservoir)	2:1	2:1	2:1
Volume of reservoir (µl)	500	80	80
Cryoprotectant solution	0.1 *M* Tris–HCl pH 8.0, 1.3 *M* ammonium sulfate, 20%(*v*/*v*) glycerol	0.1 *M* HEPES pH 7.0, 1.0 *M* succinic acid, 1%(*w*/*v*) PEG 2000 MME, 20%(*v*/*v*) propylene glycol	1.4 *M* sodium/potassium phosphate pH 7.0, 22.5%(*v*/*v*) glycerol

**Table 3:** Data collection and processing Values in parentheses are for the outer resolution shell.

	LscV_C_	LscV_C_–YscX_32_–YscY	AscV_C_–AscX_31_–YscY
Beamline and diffraction source	P14, DESY	ID23-1, ESRF	ID30B, ESRF
Wavelength (Å)	0.9763	0.9763	0.9763
Temperature (K)	100	100	100
Detector	EIGER 16M	PILATUS 6M	PILATUS3 6M
Crystal-to-detector distance (mm)	614.0	985.7	801.6
Rotation range per image (°)	0.2	0.1	0.1
Total rotation range (°)	360	360	170
Space group	*P*2_1_2_1_2	*C*222_1_	*C*222_1_
*a*, *b*, *c* (Å)	106.27, 154.29, 252.75	138.49, 372.64, 324.65	112.65, 396.23, 327.53
α, β, γ (°)	90, 90, 90	90, 90, 90	90, 90, 90
Wilson *B* factor (Å^2^)	174.14	369.99	381.47
Mosaicity (°)	0.169	0.147	0.224
Resolution range (Å)	49.28–3.75 (3.85–3.75)	49.94–7.00 (7.18–7.00)	48.45–6.96 (7.14–6.96)
Total No. of measured reflections	568961 (31004)	177068 (12458)	66676 (4661)
No. of unique reflections	43335 (3116)	13585 (982)	11932 (846)
Completeness (%)	99.8 (98.8)	99.5 (98.9)	99.4 (99.4)
Multiplicity	13.1 (9.9)	13.0 (12.7)	5.6 (5.5)
Mean *I*/σ(*I*)	14.9 (0.5)	5.44 (0.60)	7.7 (1.0)
CC_1/2_	100.0 (29.9)	99.5 (45.4)	99.5 (40.0)
*R*_meas_	0.095 (5.943)	0.291 (3.569)	0.197 (2.391)
