# Supplementary material for: Crystals of SctV from different species reveal variable symmetry for the cytosolic domain of the type III secretion system export gate
Source: Acta Crystallogr F Struct Biol Commun. 2022 Oct 14;78(Pt 11):386–94. doi: 10.1107/S2053230X22009736 (PMC9629515; doi:10.1107/S2053230X22009736)
Supplement: Supplementary file 1 [file f-78-00386-sup1.pdf]

Acta Cryst F

STRUCTURAL BIOLOGY COMMUNICATIONS

Volume 78 (2022)

Supporting information for article:

Crystals of SctV from different species reveal variable symmetry for the cytosolic domain of the type III secretion system export gate

Dominic Gilzer, Eileen Baum, Nele Lieske, Julia L. Kowal and Hartmut H. Niemann

Table S1 **.** MR solutions for $LscV_C$ produced by *Phaser*.

A monomer of $YscV_C$ was used as a search model (PDB: 7ALW).

	**Search for 8 copies**				**Search for 9 copies, solution #1**				**Search for 9 copies, solution #2**			
#Copy	Euler angles			**TFZ**	Euler angles			**TFZ**	Euler angles			**TFZ**
1	175.4	75.0	216.6	**10.3**	175.5	74.9	216.7	**10.3**	175.3	74.9	216.8	**10.3**
2	165.7	78.9	263.0	**14.1**	165.6	79.0	263.0	**14.1**	165.6	78.9	263.0	**14.1**
3	188.5	79.2	173.7	**18.3**	188.4	79.3	173.8	**18.5**	188.4	79.3	173.7	**18.5**
4	186.4	101.5	82.6	**19.2**	186.4	101.5	82.6	**19.3**	186.6	101.4	82.8	**19.3**
5	344.2	99.8	352.5	**19.8**	344.0	99.9	352.7	**19.8**	344.4	99.8	352.5	**19.8**
6	340.5	88.1	307.2	**19.6**	340.6	88.2	307.2	**19.7**	340.6	88.2	306.9	**19.7**
7	189.9	90.0	128.2	**22.6**	189.7	90.1	128.1	**22.8**	189.8	90.0	128.1	**22.8**
8	353.7	105.6	37.5	**26.6**	353.5	105.5	37.4	**25.9**	353.7	105.6	37.5	**25.9**
9					351.5	147.6	71.5	**5.7**	71.8	51.4	324.4	**-**

Table S2 Anisotropy analysis of the diffraction data performed with the STARANISO server operated by Global Phasing Ltd. (https://staraniso.globalphasing.org/cgi-bin/staraniso.cgi).

The table gives the diffraction limits (in Å) and the principal axes of the ellipsoid fitted to the diffraction cutoff surface.

	*a**	*b**	*c**
$YscV_C$:$YscX_{32}$:YscY<br>PDB ID: 7qij<br>Diffraction images: https://data.sbgrid.org/dataset/907/	4.907	3.895	3.851
$LscV_C$	4.230	4.015	3.625
$LscV_C$:$YscX_{32}$:YscY	9.567	6.710	6.232
$AscV_C$:$AscX_{31}$:YscY	6.921	6.847	6.801

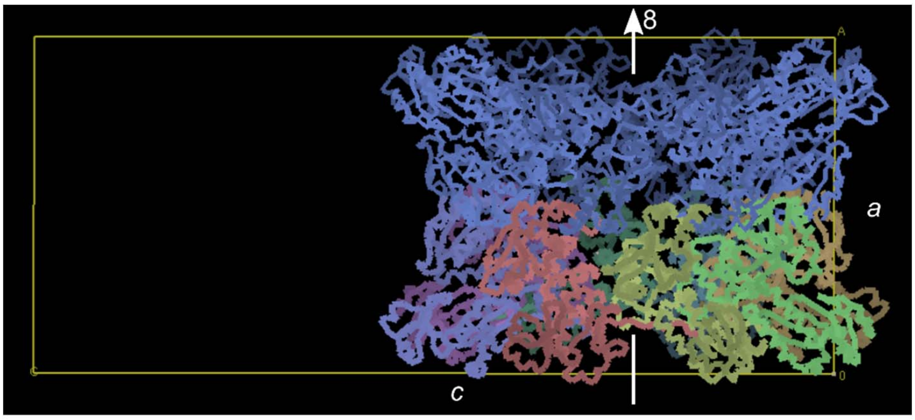

(a)

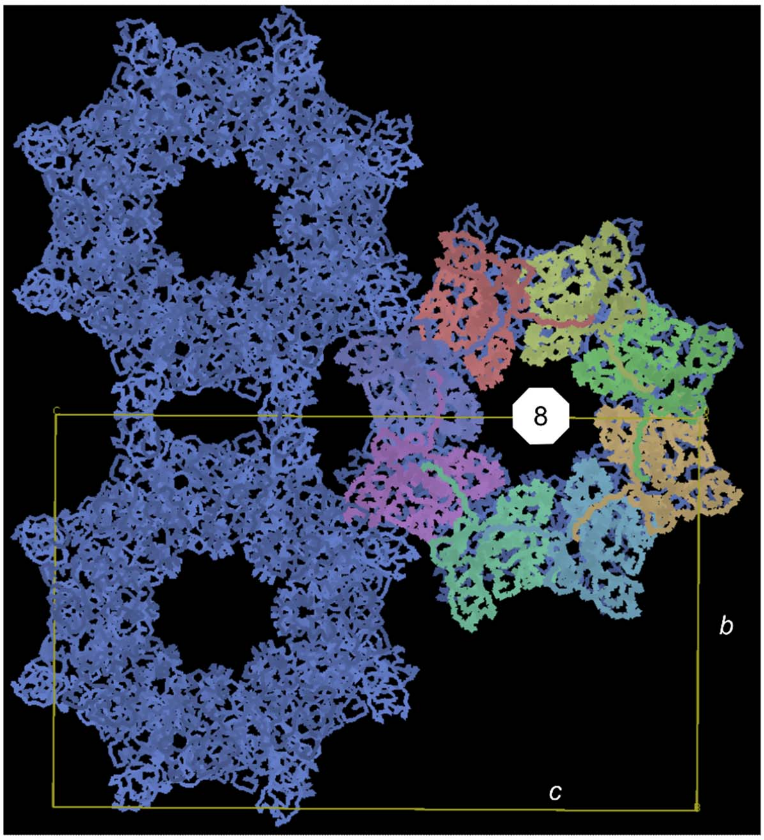

(b)

Figure S1 Stacked octameric LscV$_C$ rings in the crystal after MR. (*a*) View along the *b* axis reveals that the 8-fold rotational symmetry of LscV$_C$ runs parallel to the crystallographic 2-fold *a* axis. The rings are stacked via their membrane-distal sides. (*b*) View along the *a* axis shows co-planarity of the octamers with the *bc* plane. Close symmetry-related molecules are shown.

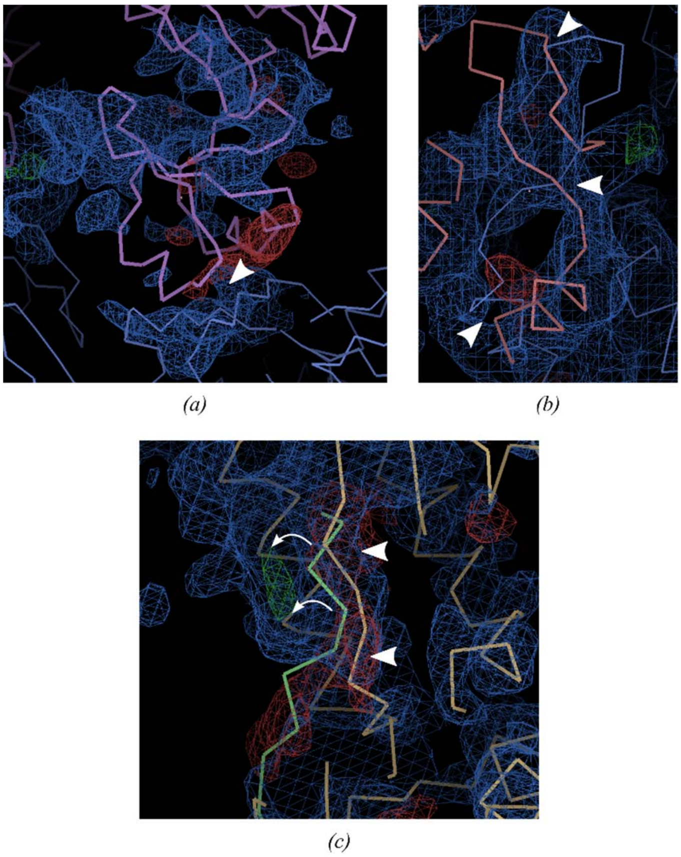

Figure S2 Packing of LscV$_{C}$ in the crystal results in clashes indicated as arrowheads. (*a*) Too-close contacts between two adjacent octameric rings (magenta and light blue) occur at the periphery of the ring. SD2 (magenta) exhibits weakly defined electron density, indicating flexibility or wrong placement. (*b*) Clashes between stacked octamers occur at the SD4-SD4 interface (orange and light blue). (*c*) The N-terminal linker of one protomer (green) folds onto the neighboring subunit (yellow). Clashes could be alleviated by moving the linker into density on the left.

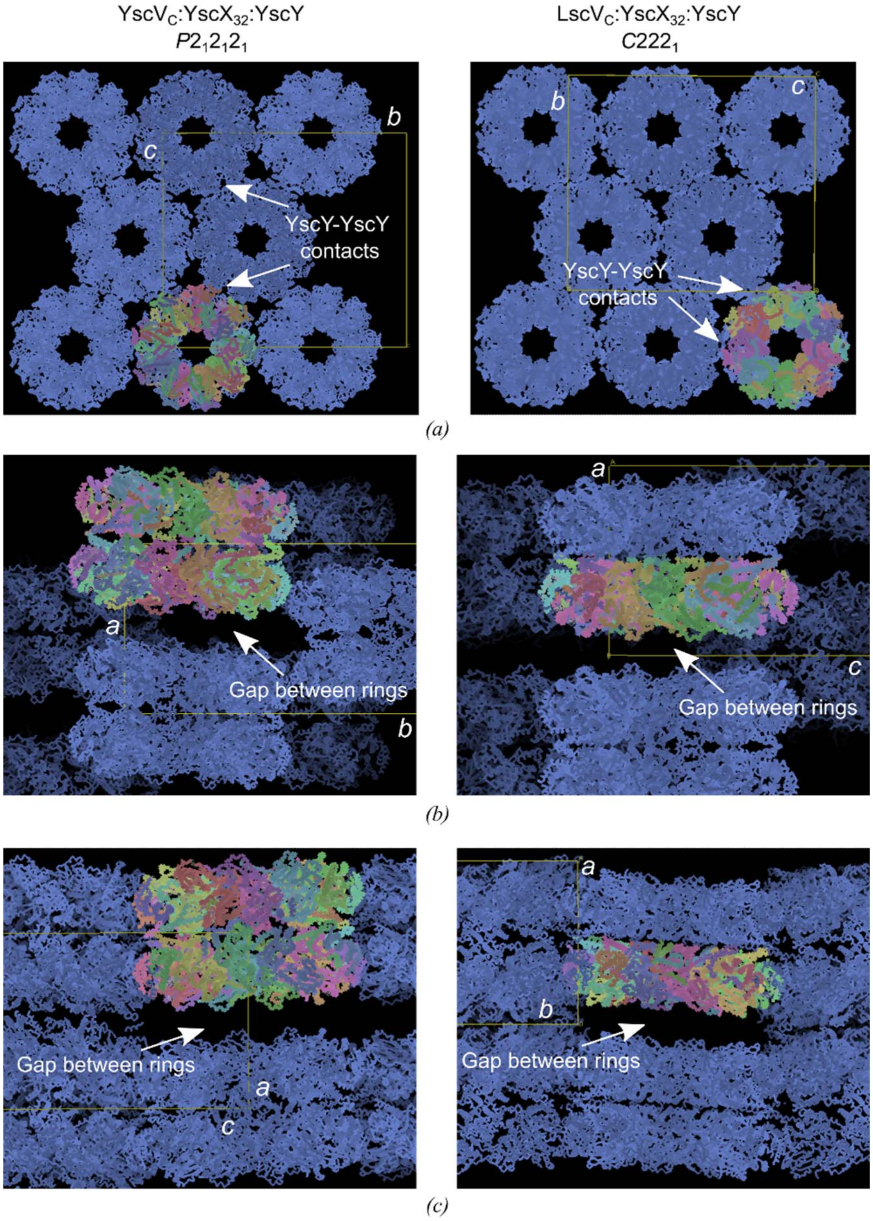

Figure S3 Packing of $YscV_C$:$YscX_{32}$:YscY (left) compared to $LscV_C$:$YscX_{32}$:YscY (right). Views along the (*a*) shortest, (*b*) middle, and (*c*) longest axis of the unit cell. Crystal contacts are almost entirely made between two molecules of YscY on the periphery of the nonameric ring. Along the *a* axis, stacked nonamers are separated by larger solvent channels. Images of $YscV_C$:$YscX_{32}$:YscY reprinted from Gilzer *et al.*, 2022.

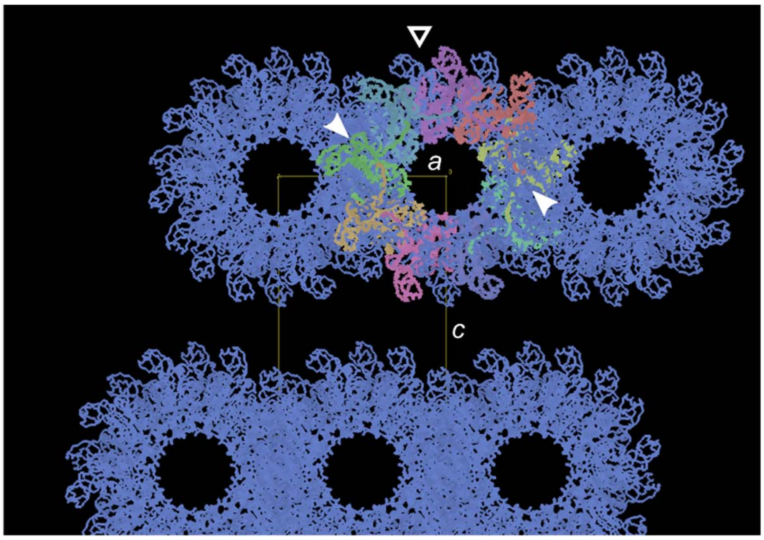

(a)

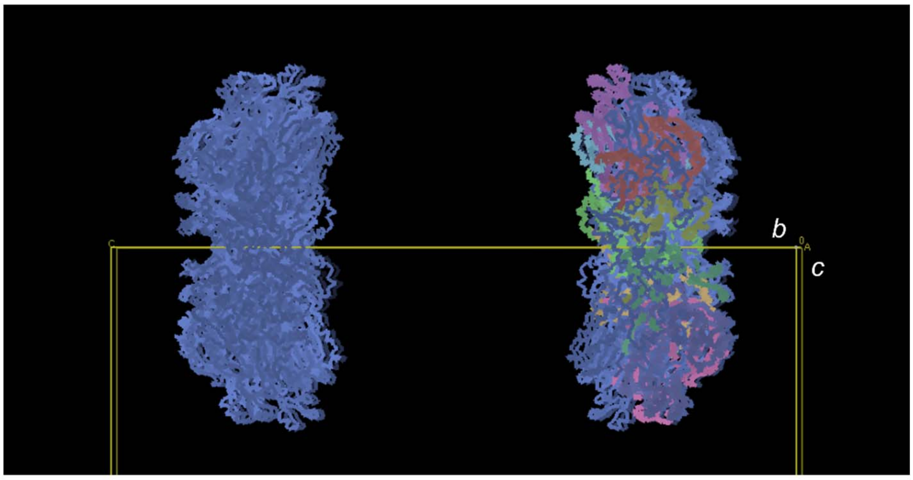

(b)

Figure S4 Wrong MR solution for AscV$_C$:AscX$_{31}$:YscY produced when searching with a YscV$_C$ nonamer (PDB: 7ALW) as model. (*a*) View along the *b* axis shows large gaps along *c* and severe clashing of nonameric rings. Adjacent oligomers overlap with more than one protomer (white arrowheads) and a symmetry-related ring overlaps almost entirely (triangle). (*b*) View along the *a* axis presents large gaps between the placed model and a symmetry mate along the *b* axis.
